# When pseudo–Richter transformation hits the spleen: a case report and literature review

**DOI:** 10.1007/s12308-026-00704-7

**Published:** 2026-06-02

**Authors:** Ricard Onieva, Laura Rama, Carmen Blázquez, Rut Astorga, Laura Escudero, Maria Elena Ramila, M. Carmen Ramos, Natalia Papaleo

**Affiliations:** 1 Department of Pathology, Universitat Autònoma de Barcelona, Sabadell, Spain; 2 Hematology Department, Universitat Autònoma de Barcelona, Sabadell, Spain

**Keywords:** Chronic lymphocytic leukemia, Pseudo-Richter, Splenic rupture, Ibrutinib

## Abstract

Chronic lymphocytic leukemia/small lymphocytic lymphoma (CLL/SLL) is the most prevalent leukemia in adults which typically follows an indolent clinical course. However, it has the potential to transform into more aggressive categories, including accelerated CLL or diffuse large B-cell lymphoma (DLBCL), a phenomenon known as Richter transformation. Bruton tyrosine kinase inhibidors (BTKI), such as ibrutinib and zanubrutinib, represent a cornerstone of CLL/SLL treatment by inhibiting B-cell receptor signaling. Temporary discontinuation of these agents are common due to surgical procedures or infections which has been associated with rapid disease progression, producing a pseudo–Richter transformation (P-RT) in tissues. We present a case with CLL/SLL who experienced clinical deterioration following ibrutinib withdrawal, resulting in an explosive disease flare and splenic rupture. The patient improved after BTKI reintroduction, and a P-RT was diagnosed. Nine similar patients were found through PubMed search, and we describe their clinicopathological characteristics.

## Introduction

Chronic lymphocytic leukemia/small lymphocytic lymphoma (CLL/SLL) is the most common low-grade B-cell leukemia in adults and typically follows an indolent clinical course. However, it has the potential to transform into more aggressive categories, including accelerated CLL (aCLL) or a high-grade lymphoproliferative disorder. The most frequent transformation is to diffuse large B-cell lymphoma (DLBCL), a phenomenon known as Richter transformation (RT) [1, 2]. RT is an uncommon event and usually occurs in CLL/SLL cases with high-risk biological features, including complex karyotype, unmutated immunoglobulin heavy chains (IGHV) status, *TP53* gene aberrations, and *NOTCH1* mutations [3, 4].

Bruton tyrosine kinase inhibitors (BTKIs), such as ibrutinib and zanubrutinib, represent a cornerstone of CLL/SLL treatment by inhibiting B-cell receptor signaling. Temporary interruptions in treatment are common, due to surgical procedures or serious infections. This discontinuation has been associated with rapid disease progression, manifested by increased lymphadenopathy, lymphocytosis, and B symptoms [5]. In this setting, some patients develop histological findings characterized by large transformed cells that are morphologically and immunophenotypically compatible with DLBCL. Nevertheless, these cases do not represent true RT, as the lymphomatous component regresses after reintroduction of BTKI therapy, preserve the expression of CD23, LEF1 and generally do not harbor *MYC* rearrangement [6–8]. This phenomenon, termed pseudo-Richter transformation (P-RT), is thought to reflect an accelerated CLL rather than overt DLBCL. In contrast, true RT is refractory to BTKI therapy and requires aggressive immunochemotherapy, most commonly R-CHOP like regimens, associated with a dismal prognosis [9].

Moreover, aCLL commonly mimics RT with aggressive clinical behavior, making the distinction between these entities also challenging. Its diagnosis is mainly based on histological findings including expanded proliferation centers with the absence of confluent diffuse sheets of large cells, an increased proliferative rate (Ki67 > 40%) and mitotic activity (> 2.4 mitosis) [1].

We present a patient with CLL/SLL who experienced clinical deterioration following ibrutinib withdrawal, resulting in an explosive disease flare and splenic rupture. The patient improved after BTKI reintroduction and a P-RT was diagnosed. Nine similar cases of P-RT were found through PubMed search none with splenic rupture. We describe their clinicopathological characteristics.

## Clinical history

A 74-year-old male was admitted to our hospital due to diarrhea. His medical history was notable for chronic lymphocytic leukemia/small lymphocytic lymphoma (CLL/SLL), diagnosed 17 years ago. He was well controlled with ibrutinib for the past 5 years, as a second line after rituximab-fludarabine-cyclophosphamide regimen. However, due to intercurrent medical conditions, treatment had been temporarily discontinued on several occasions. Each interruption was followed by a marked increase in leukocyte count.

Because of the current episode of diarrhea and pneumonia, ibrutinib was again discontinued. Eight days after treatment withdrawal, an abdominal CT scan revealed progression of supra- and infradiaphragmatic lymphadenopathy, along with splenic rupture and hemoperitoneum. The patient underwent an urgent splenectomy, and the specimen was submitted to the pathology department for further evaluation.

## Materials and methods

Histological examination of the spleen was carried out and immunohistochemical studies were performed on formalin-fixed paraffin-embedded tissue sections. Tissue sections were stained with hematoxylin and eosin, CD20 (L26, Ventana), CD3 (Polyclonal, Dako), CD5 (EP204, DBS), CD23 (DAK-CD23, Dako), LEF-1 (EPR2029Y, ABCAM), CD10 (DAK-CD10, Dako), p53 (DO-7, Dako), BCL-2 (124, Ventana), BCL-6 (PG-B6p, Dako), ki67 (30_9, Ventana), cMYC (EP121, Biocare), and LMO2 (SPr1, Cell Marque). FISH was also ordered to identify rearrangements of *MYC*,* BCL2*, and *BCL6* genes, using probes by Vysis, Abbott Molecular, and Des Plaines city, IL, USA.

## Results

Histopathological examination of the spleen revealed partial architectural effacement by a diffuse proliferation of small- to intermediate-sized lymphocytes expanding the white pulp and infiltrating the red pulp. The neoplastic cells showed scant cytoplasm, round nuclei, and clumped chromatin. Immunohistochemically, they were positive for the B-cell marker CD20, with aberrant expression of CD5, CD23, and LEF1. A post-germinal center phenotype with MUM-1 positive cells and p53 expression were also observed. CD10, BCL6, and MYC were negative. These morphological and immunophenotypic findings were consistent with a diagnosis of CLL/SLL.

**Fig. 1:**
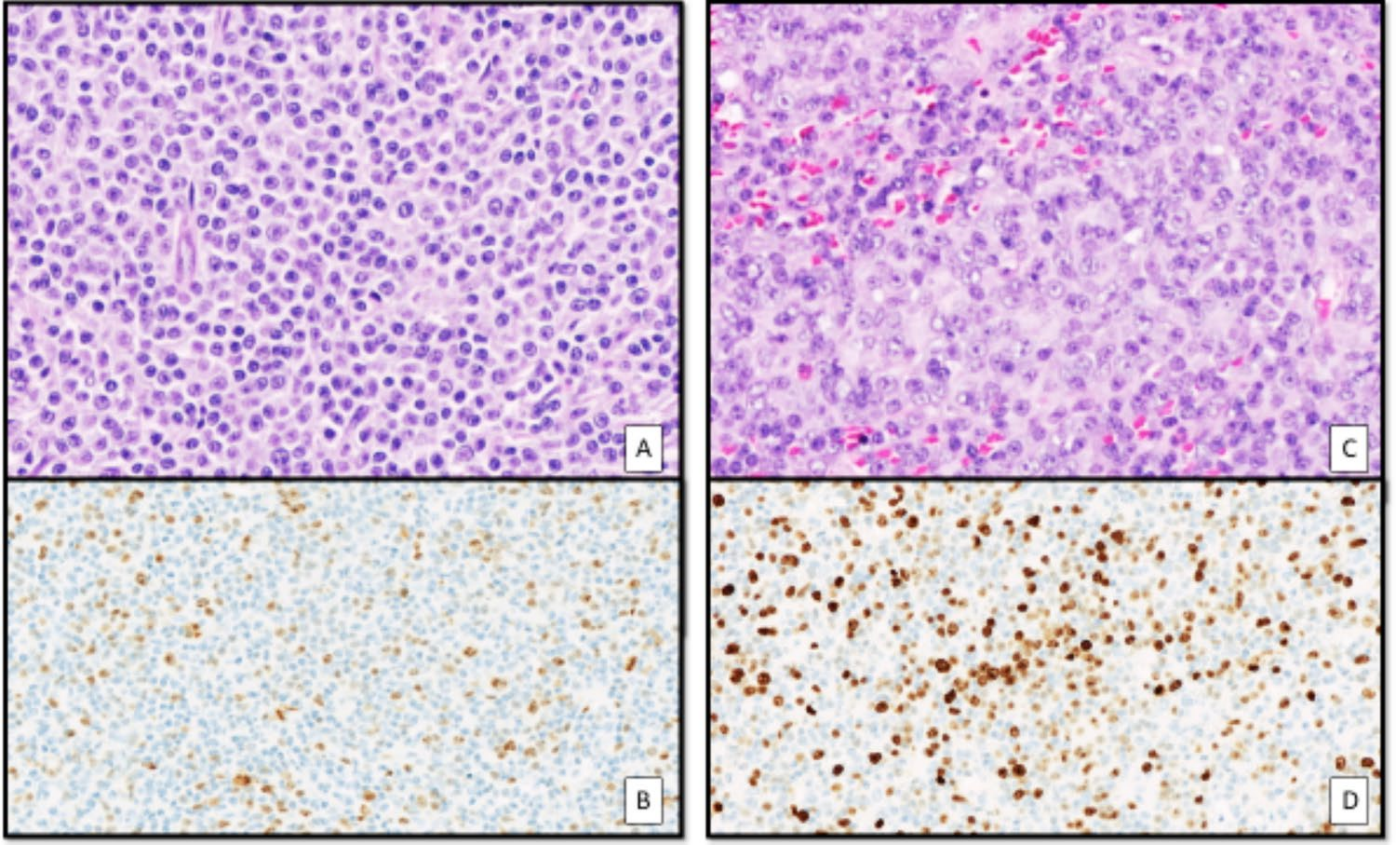
**A**,** B** Hematoxylin and eosin staining showing diffuse proliferation of small- to intermediate-sized lymphocytes with a low proliferative index (Ki-67) (images taken at × 200). **C**,** D** Hematoxylin and eosin staining showing clusters of large transformed lymphoid cells, characterized by vesicular chromatin and prominent nucleoli, with a high proliferative index (Ki-67) (images taken at × 100)

However, clusters of large transformed lymphoid cells involving more than 20% of the splenic infiltrate were identified, characterized by irregular nuclear contours, vesicular chromatin, and prominent nucleoli, including paraimmunoblasts and prolymphocytes. No significant immunophenotypic differences were observed between the two components, except for a heterogeneous proliferation index: the small-cell component showed a Ki-67 index of approximately 5%, whereas the larger transformed cells demonstrated a higher proliferation rate, reaching up to 40% (Fig. 1). The peripheral blood analysis revealed pathogenic NOTCH1 p.(Pro2514Argfs*4) mutation (allele frequency 50.2%), *XPO1 p.(Glu571Lys)* mutation (allele frequency 37.5%), and *TP53 p.(Val143Met)* mutation (allele frequency 14.1%), which was not present two years before. These findings supported a diagnosis of P-RT with areas of aCLL, rather than a RT.

Treatment was subsequently switched to zanubrutinib, resulting in a marked improvement in leukocyte count, which decreased from 126.57 to 12.66 × 10^9^/L. Three months later, the patient was readmitted with pulmonary thromboembolism and seizures. Computed tomography revealed findings consistent with a central nervous system infection, and despite appropriate treatment, the patient died.

## Discussion

Ibrutinib is a highly effective and widely used therapy in CLL/SLL, producing durable clinical responses across disease settings. Nevertheless, treatment discontinuation, whether driven by disease progression or therapy-related toxicity, is frequent and confers a substantial risk of disease flare, highlighting the need for vigilant clinical management during therapy transitions [5].

In 2020, Slonim et al. first introduced the term P-RT syndrome to describe a phenomenon observed following ibrutinib withdrawal, highlighting a clinically important mimic of true RT [6].

A review of the English-language literature identified nine reported cases of P-RT, all of which share demographic and genetic characteristics similar to those observed in our case (Table 1).

**Table 1:** Pseudo-Richter transformation cases described in the literature

Reference	Age (years)	Sex	Site of P-RT diagnosis	Treatment	Withdrawal period (days)	Cytogenetics results	*TP53* status	IgVH	*MYC* FISH result
Shi [8]	Advanced	Male	LN, LNE	Acalabrutinib	3	NA	NA	NA	NA
Hampel [7]	73	Male	Mesenteric LN	Ibrutinib	7	del17p, + 12, del13q	Mutated (NGS)	Not mutated	Not rearranged
Hampel [7]	63	Male	Mesenteric, LN	Ibrutinib	7	del17p, del13q	Mutated (NGS)	Not mutated	Not rearranged
Hampel [7]	44	Male	Inguinal LN	Ibrutinib	2	12	Mutated (NGS)	Not mutated	Not rearranged
Slonim [6]	69	Male	LN, LNE	Ibrutinib	7	+ 12, 13q-, 11q-, 17p-	Mutated (17p-)	Not mutated	Not rearranged
Slonim [6]	82	Male	LN, LNE	Ibrutinib	10	17p-	Mutated (17p-)	NA	Not rearranged
Slonim [6]	66	Male	LN, LNE	Ibrutinib	7	12	NA	Not mutated	Not rearranged
Slonim [6]	83	Male	Bone marrow	Ibrutinib	10	+ 12, 11q-	NA	NA	Not rearranged
Slonim [6]	70	Female	LN, LNE	Ibrutinib	13	+ 12, 13q-	NA	NA	Not rearranged
Current case	74	Male	Spleen	Ibrutinib	10	del17p, del13q	Mutated (NGS)	Not mutated	Not rearranged

Overview of reported pseudo-Richter transformation cases

*LN* lymph node, *LNE* location not specified, *NA* not available

Notably, all published cases share the presence of a *TP53* mutation, unmutated IGHV status, and the absence of *MYC* rearrangement, as was detailed in eight cases of the 2024 EA4HP/SH lymphoma workshop [9]. These features may be useful in differentiating P-RT from true RT [6–8].

In our case, histological examination of the spleen revealed aggregates of large transformed lymphoid cells with DLBCL-like morphology involving more than 20% of the infiltrate, whereas the remaining areas retained the appearance of underlying CLL. These cells displayed irregular nuclear contours, vesicular chromatin, and prominent nucleoli, consistent with paraimmunoblasts and prolymphocytes, with a proliferative index of approximately 40%, lower than that typically encountered in bona fide RT [10] and differs from the more diffuse large-cell transformation described in previously reported cases. A similarly low proliferative index was reported by Min et al., although the patient in that case was asymptomatic [8]. In contrast, our patient experienced an aggressive disease flare culminating in splenic rupture. Considering the close temporal relationship with recent ibrutinib withdrawal and the marked clinical acceleration observed, we propose that aCLL with focal large-cell transformation in this context may represent a clinicopathologic equivalent within the spectrum of P-RT.

Currently, CLL/SLL, aCLL, and RT are thought to represent a spectrum of the same disease, making it plausible to identify intermediate forms [11, 12]. In this context, our findings raise the possibility that this case represents a true P-RT with aCLL-like morphology, reflecting the intermediate morphological features observed.

## Conclusions

We report a patient with CLL/SLL who experienced clinical deterioration following ibrutinib withdrawal, with an explosive disease flare resulting in splenic rupture. To our knowledge, this is the first reported case of splenic rupture associated with P-RT with aCLL-like morphology. Importantly, the patient improved after reintroduction of a BTKI therapy.

Recognition of P-RT by both clinicians and pathologists is essential to prevent premature discontinuation of effective BTKI therapy and to avoid unnecessary morbidity associated with intensive immunochemotherapy. This underscores the importance of integrating comprehensive clinical data to ensure accurate diagnosis and optimize management of this complication.

## Data Availability

No datasets were generated or analysed during the current study.
